# Temperatures Rising: Sprawling Cities Have the Most Very Hot Days

**DOI:** 10.1289/ehp.118-a444a

**Published:** 2010-10

**Authors:** Tanya Tillett

**Affiliations:** **Tanya Tillett**, MA, of Durham, NC, is a staff writer/editor for *EHP*. She has been on the *EHP* staff since 2000 and has represented the journal at national and international conferences

The urban heat island effect, the phenomenon in which a city has higher temperatures than surrounding countryside, is known to contribute to higher rates of heat-related mortality in summer months when temperatures soar. Although extreme heat events have become more common in large U.S. cities, a new study indicates that sprawling cities experience more than double the rate of extreme heat events in the summer compared with more compact urban areas [*EHP* 118(10):1425–1428; Stone et al.].

The authors considered 53 U.S. metropolitan areas for which data were available on sprawl and extreme heat event. Sprawl had been assessed in a 2003 study using an index based on land use data from the 2000 census along with measures of population density, average street block size, proximity of homes to businesses, and land use mix. Extreme heat events were defined as days on which the minimum, maximum, or average temperature exceeded the 85th percentile of a base period of 1961–1990.

The authors compared the extent of sprawl for each metropolitan area with that area’s average annual rate of extreme heat events from 1956 through 2005. Over the 50 years examined, the number of extreme heat events increased by an average of 14.8 days in cities with the most sprawl (e.g., Atlanta, Tampa, and Grand Rapids), whereas more compact cities (e.g., Chicago, Boston, and Baltimore) saw a lower average increase of 5.6 days of very high temperatures. The observed connection between extreme heat and sprawl was independent of climate zone and variations in size and growth of metropolitan populations. However, data analysis indicated that between 1992 and 2001 the deforestation rate in the most sprawling areas was more than double that of the most compact cities.

The study did not allow for an examination of the differences in rates of heat-related morbidity and mortality between sprawling and compact cities. However, the authors write that the numerous adverse effects associated with urban sprawl (e.g., high levels of ozone, poor water quality, and decreased physical activity) signal a need for public health officials to adopt more risk-reduction strategies such as preserving regional green space, installing green roofs, and replacing vehicular traffic with more public-transit options. They note that many of these strategies can also increase urban resilience to other climate-related risks including increased severe precipitation.

